# Visceral leishmaniasis treatment outcome and its determinants in northwest Ethiopia

**DOI:** 10.4178/epih.e2017001

**Published:** 2016-12-28

**Authors:** Getachew Mebrahtu Welay, Kefyalew Addis Alene, Berihun Assefa Dachew

**Affiliations:** 1 Aksum University, Shire Campus, Shire, Ethiopia; 2 Department of Epidemiology and Biostatistics, Institute of Public Health, College of Medicine and Health Sciences, University of Gondar, Gondar, Ethiopia; 3 The University of Queensland, School of Public Health, Herston Qld 4006, Australia

**Keywords:** Visceral leishmaniasis, Treatment outcome, Determinants, Ethiopia

## Abstract

**OBJECTIVES:**

Poor treatment outcomes of visceral leishmaniasis (VL) are responsible for the high mortality rate of this condition in resource-limited settings such as Ethiopia. This study aimed to identify the proportion of poor VL treatment outcomes in northwest Ethiopia and to evaluate the determinants associated with poor outcomes.

**METHODS:**

A hospital-based retrospective study was conducted among 595 VL patients who were admitted to Kahsay Abera Hospital in northwest Ethiopia from October 2010 to April 2013. Data were entered into Epi Info version 7.0 and exported to SPSS version 20 for analysis. Bivariate and multivariate logistic regression models were fitted to identify the determinants of VL treatment outcomes. Adjusted odds ratio (aORs) with 95% confidence intervals (CIs) were used, and *p*-values <0.05 were considered to indicate statistical significance.

**RESULTS:**

The proportion of poor treatment outcomes was 23.7%. Late diagnosis (≥29 days) (aOR, 4.34; 95% CI, 2.22 to 8.46), severe illness at admission (inability to walk) (aOR, 1.63; 95% CI, 1.06 to 2.40) and coinfection with VL and human immunodeficiency virus (HIV) (aOR, 2.72; 95% CI, 1.40 to 5.20) were found to be determinants of poor VL treatment outcomes.

**CONCLUSIONS:**

Poor treatment outcomes, such as death, treatment failure, and non-adherence, were found to be common. Special attention must be paid to severely ill and VL/HIV-coinfected patients. To improve VL treatment outcomes, the early diagnosis and treatment of VL patients is recommended.

## INTRODUCTION

Visceral leishmaniasis (VL) is the most severe vector-borne protozoan disease caused by *Leishmania donovani*, and is transmitted by the bite of female *Phlebotomus* sand flies [1,2]. VL is a global health problem, imposing an estimated disease burden of 1.98 million disability-adjusted life years and 20,000 to 40,000 deaths per annum around the world [1].

Ninety percent of the global burden of VL occurs in India, Bangladesh, Brazil, Sudan, South Sudan, and Ethiopia [3-5]. Although VL is treatable, in sub-Saharan Africa, mortality associated with VL is still high. This is directly or indirectly related to poor treatment outcomes, which occur for a number of reasons [6,7].

Ethiopia is a country highly affected by VL, with 1,860 cases of VL reported annually and an estimated annual incidence of VL ranging from 3,700 to 7,400 cases [8].

In Ethiopia, the northwest lowland areas (Metema and Humera districts) bordering Sudan are the most important endemic foci of VL, accounting for 60% of the VL burden in the country [9,10]. This region also has the highest burden of VL/human immunodeficiency virus (HIV) coinfection in the world, with the HIV prevalence among VL patients ranging from 19 to 41% [10].

Poor treatment outcomes for VL have become a challenging public health problem in Ethiopia, particularly in the northwest region of the country. One of the greatest concerns regarding VL is its high fatality rate, which reaches almost 100% among non-treated symptomatic patients, in contrast to a 10% fatality rate among VL patients who undergo treatment.

Data on treatment outcomes and the determinants thereof would enable policymakers, clinicians, and funding organizations to establish priorities for addressing VL incidence and mortality in the northwestern sub-region of Ethiopia. However, although there have been a few studies on VL/HIV coinfection, the proportion of poor treatment outcomes among VL patients and the determinants of treatment failure have not been well investigated in the study area. Hence, this study aimed to assess the proportion and determinants of poor treatment outcomes among VL patients at Kahsay Abera Hospital in northwest Ethiopia.

## MATERIALS AND METHODS

### Study design and setting

A hospital-based retrospective study was conducted at Kahsay Abera Hospital in northwestern Ethiopia. This hospital is a general facility where all diseases are treated. There is a leishmaniasis treatment center within the hospital, which is located in Humera, one of the most important VL endemic foci in the country. Humera, at an elevation of 600 m above sea level, is characterized by a very harsh environment, with an annual temprature ranging from 27 to 45°C and rainfall ranging from 900 to 1,800 mm per annum. Humera is one of Ethiopia’s most fertile agricultural zones, with large-scale farming of cash crops such as sesame, maize, cotton, and sorghum. Since 1970, Humera has undergone an extensive program of agricultural development, with a consequent influx of migrant workers, which has led to a rapid increase in the number of VL cases. Migrants travel to Humera to work in the sowing and harvest seasons, mainly from October to February and from May to July. Seasonal agricultural work attracts approximately 500,000 migrant laborers each year from non-endemic parts of Tigray and neighboring regions [11].

A clinical case of VL is defined as a person who presents with fever for more than 2-week and an enlarged spleen (splenomegaly) and/or enlarged lymph nodes (lymphadenopathy), or symptoms such as loss of weight, anemia, or leukopenia, while living in or having recently travelled to a known endemic area for VL. Diagnosis of VL at Kahsay Abera Hospital is performed according to the standardized Médecins Sans Frontières (MSF) and Ethiopian Ministry of Health guidelines using a positive rK39 rapid diagnostic test (DiaMed-IT-Leish, DiaMed, Cressier, Switzerland) and the microscopic examination of aspirates from the spleen [12]. The diagnosis has a high specificity, but the sensitivity of the microscopy varies (93 to 99% for the spleen, 53 to 86% for bone marrow, and 53 to 65% for lymph node aspirate) [13].

Treatment is normally provided only after the presence of the disease is confirmed based on a clinical examination and labora2tory tests. Sodium stibogluconate monotherapy is administered through intramuscular injections of 20 mg/kg/d for 30 days. The treatment regimen at the hospital follows the national guidelines.

### Definitions of terms in visceral leishmaniasis cases

An initial cure was defined as a patient showing improvement in signs and symptoms after 30 days of standard treatment (fever resolution, hemoglobin increase, weight gain, and spleen size regression), the absence of parasites in smears, and a negative test-of-cure (TOC) culture. In the current study, we were not able to assess whether the cure was definitive at a 6-month follow-up examination, since VL patients are often migrants from rural communities and are difficult to trace once they are discharged from the hospital. Poor treatment outcomes were defined as death, treatment failure, and non-adherence. Initial treatment failure was defined as a positive TOC culture (parasitological failure) and/or clinical signs/symptoms that persisted after 30 days of standard treatment. Functional status was defined as an individual’s ability to perform the normal daily activities required to meet basic needs, fulfill his or her usual roles, and maintain health and well-being.

### Inclusion and exclusion criteria

All VL patients at the Kahsay Abera Hospital Leishmaniasis Treatment Center who underwent anti-leishmaniasis treatment from October 2010 to April 2013 were included in this study. More recent data were not included, as the proper data-handling format was not completely implemented at the hospital after the MSF project was phased out. A total of 890 VL patients were registered during the study period, of whom 595 patients were included. Those who were transferred out to other health institutions for better management and those for whom the available information was incomplete were excluded from the study.

### Data collection

Demographic, clinical, immunological, and laboratory profile data were collected using a prepared data extraction tool. Four trained health professionals collected the data from the patients’ charts after confirming that the records were complete.

Treatment outcomes and patient characteristics were collected from the medical registry casebook and VL patient charts, respectively, and transferred to the data extraction tool after the completion of 30 days of standard treatment.

A good treatment outcome was considered to be cure of the disease as measured by both a laboratory test (a negative TOC culture at the end of 30 days of standard treatment) and clinical signs and symptoms (weight gain, normal body temperature, and regressed spleen). Non-adherence (patients who were lost to follow-up or did not pick up their drugs for more than 15 days), treatment failure (non-response or failure to decrease the parasitological grade after a 30-day course of the treatment regimen) and death due to visceral leishmaniasis were considered to be poor treatment outcomes [14].

### Data analysis

Data were cleaned and entered into a computer using Epi Info version 7 (Centers for Disease Control and Prevention, Atlanta, GA, USA) and exported to SPSS version 20 (IBM Corp., Armonk, NY, USA) for analysis. Both descriptive and analytical statistical procedures were utilized. Descriptive statistics such as percentages, mean values, and standard deviations were used to present our results. Binary logistic regression was used to identify factors associated with poor treatment outcomes among VL patients. Bivariate analysis was performed to identify the association of each independent variable with VL treatment outcomes. Variables with p-values <0.2 in the bivariate analysis were entered into the multivariate analysis to identify the determinants of poor outcomes while controlling for the possible effect of confounds. Adjusted odds ratios (aORs) with 95% confidence intervals (CIs) were used to determine the strength of associations, and variables with a p-value <0.05 in the final model were taken as significant determinants of treatment outcomes. The goodness-of-fit of the model was tested using the Hosmer–Lemeshow test for the full model (p>0.05).

### Ethics approval and consent to participate

Ethical clearance was obtained from the Ethical Review Committee of the Institute of Public Health, University of Gondar. A permission letter was also obtained from the Tigray Regional State Health Bureau to review pre-existing health records. To ensure confidentiality, patients’ names were not extracted from the records.

## RESULTS

### Demographic characteristics

A total of 595 VL patient records were included in the analysis. The patients’ mean age was 25.9±9.6 years, and a majority of them (535, 89.9%) were in the age group of 16 to 45 years. Almost half of them (284, 47.7%) were residents of the district, and one-third (220, 37.0%) were migrants from nearby districts and zones. The majority of patients (555, 93.3%) were males (Table 1).

### Baseline clinical and laboratory profiles of the patients

The mean time between clinical manifestations and hospital admission was 25.82 days, ranging from 2 to 365 days. One-fourth of VL patients (141, 23.7%) had a delay in diagnosis and treatment. The mean hospital stay of patients during treatment was 23.6±10.5 days. The mean hemoglobin level of patients was 8.98±4.26 g/dL (Table 2).

### Determinants of poor visceral leishmaniasis treatment outcomes

The proportion of poor VL treatment outcomes was found to be 23.7%, of which 12.4% were death, 5.7% were treatment failure, and 5.6% were non-adherence.

Delayed diagnosis and treatment, inability to walk at hospitalization, baseline platelet count, and tuberculosis and HIV coinfections were found to be significantly associated with poor VL treatment outcomes in the univariate analysis.

However, in the multivariate logistic regression analysis, only delayed diagnosis and treatment, inability to walk at hospitalization, and VL/HIV coinfection remained significantly and independently associated with poor VL treatment outcomes.

VL patients who were admitted to the treatment center late (≥29 days after the onset of symptoms) were 4.34 times more likely to have poor treatment outcomes than patients who were admitted early (<13 days) (aOR,4.34; 95% CI, 2.22 to 8.46).

VL patients who were unable to walk (severely ill) at admission were at a 1.63 times greater risk of poor treatment outcomes than those who were able to walk (moderately ill) (aOR, 1.63; 95% CI, 1.06 to 2.40). Similarly, HIV coinfected VL patients were at a 2.72 times greater risk of poor treatment outcomes than non-coinfected VL patients (aOR, 2.72; 95% CI, 1.40 to 5.20) (Table 3).

## DISCUSSION

Copious data on treatment outcomes and their determinants among VL patients are necessary for national control, prevention, and elimination of VL.

In comparison with other similar studies conducted elsewhere in Ethiopia (18.5%) [15], poor VL treatment outcomes were found to be common (23.7%) in the current study, which was conducted in northwest Ethiopia. This proportion of poor outcomes is also higher than that found in a study conducted in Brazil (2%) [16]. This could be due to differences in design, setting, and the type of subjects involved in the study.

However, the proportion found in this study was lower than that found in a study conducted in another part of Ethiopia (31.6%) [17]. This discrepancy may be due to differences in the study subjects, as the previous study was performed on HIV-VL coinfected patients, and VL/HIV coinfection increases the likelihood of poor treatment outcomes. However, the results of the present study correspond to those obtained in West Bengal, India (22.7%) [14] and Peru (24.4%) [18].

Although the current study used secondary data and was limited in the possible variables it could identify that might have had an effect on treatment outcomes, this study identified late presentation and admission to the treatment center, severe illness as measured by the functional status of patients at admission, and HIV/VL coinfection as determinants of poor VL treatment outcomes.

VL patients who were admitted to the treatment center with a delay of more than a month (≥29 days) from the onset of symptoms were four times more likely to have poor treatment outcomes than patients who presented and were admitted earlier. Delays in diagnosis and treatment are a bottleneck challenge in migrant and rural communities working in remote agricultural areas where health facilities are limited and day laborers lack knowledge and awareness of the need for early admission to a health facility after the onset of clinical symptoms. The negative effects of delays in diagnosis and treatment on outcomes have been reported in Peru [18], Georgia [19], and South Sudan [20]. This suggests a need for increased community awareness of the necessity of visiting a health institution as soon as the first clinical symptoms of VL manifest.

In addition, further analysis indicated that VL patients who were unable to walk at admission (severely ill) were 1.63 times more likely to have poor treatment outcomes as compared to those who were able to walk (moderately ill). This result is comparable to the findings of a study conducted in Brazil. This association is due to the fact that clinically deteriorated patients may come in at a critical stage, resulting in poor treatment outcomes and a poor prognosis [5].

This study also found that VL/HIV coinfection was a determinant of poor treatment outcomes, as VL/HIV-coinfected patients were 2.72 times more likely to have poor treatment outcomes than non-coinfected patients. This can be easily explained by the pathophysiology of the two diseases, as both HIV and VL lead to a compromised immune status in the patient and poor treatment outcomes [5,16,21].

This study has several limitations that should be considered when interpreting its results. The data were retrospectively extracted from patients’ medical records, and some important variables such as parasite load, total leukocyte count, and CD4 count were inadequately recorded and were not included in the analysis. Moreover, a few patients were discharged and considered cured before the full treatment course was completed and/or without a TOC culture. In addition to this, only 595 of the 890 VL patients were included in the study, while the remaining patients were excluded from the study because they were referred to other facilities or their data files were missing. This may have led to bias and underestimation or overestimation of the associations with treatment outcomes if the patient exclusion conditions were related to treatment outcomes.

Poor VL treatment outcomes, defined as death, treatment failure, or non-adherence, were found to be common among VL patients. Late presentation and admission to the treatment center, inability to walk at admission (severe illness), and HIV/VL coinfection were determinants of poor VL treatment outcomes. Hence, VL treatment outcomes would be improved if patients sought care at a health institution at the earliest manifestation of clinical symptoms, and if special attention were given to severely ill and VL/HIV-coinfected patients. In addition, prioritizing treatment for communities in which VL is endemic and VL patient education are advisable. Moreover, a prospective follow-up study design would be helpful for determining other important variables related to VL treatment outcomes.

## Figures and Tables

**Table 1.** Demographic characteristics of visceral leishmaniasis patients at Kahsay Abera Hospital, Humera, Ethiopia, 2010-2013 (n=595)

Characteristics	Frequency	%
Age (yr)		
≤15	29	4.9
16-45	535	89.9
≥46	31	5.2
Sex		
Male	555	93.3
Female	40	6.7
Residence		
Rural	400	67.2
Urban	195	32.8
Migration status		
Migrants	220	37.0
Residents	284	47.7
New settlers	91	15.3

**Table 2.** Clinical and laboratory profiles of visceral leishmaniasis patients at Kahsay Abera Hospital, Humera, Ethiopia, 2010-2013 (n=595)

Characteristics	Frequency	%		
Time elapsed before diagnosis (d)		
<13	107	18.0
14-28	347	58.3
≥29	141	23.7
General condition of patients		
Able to walk	357	60.0
Unable to walk	238	40.0
Fever		
No	28	4.7
Yes	567	95.3
Weight loss		
No	64	10.8
Yes	531	89.2
Jaundice		
No	537	90.3
Yes	58	9.7
Splenomegaly		
No	98	16.5
Yes	497	83.5
Anemia		
No	173	29.1
Yes	422	70.9
Hemoglobin (g/dL)		
<7.9	279	46.9
8.0-10.9	102	17.1
≥11.0	214	36.0
Platelet count (cells/mm^3^)		
<85,000	265	44.5
≥85,000	330	55.5
BMI (kg/m^2^)		
<18.5	511	85.9
≥18.5	84	14.1
Tuberculosis		
No	534	89.7
Yes	61	10.3
Malaria		
No	469	78.8
Yes	126	21.2
HIV status		
No	546	91.2
Yes	49	8.2

BMI, body mass index; HIV, human immunodeficiency virus.

**Table 3.** Bivariate and multivariate logistic regression analysis of the factors associated with poor treatment outcomes among visceral leishmaniasis patients at Kahsay Abera Hospital, Humera, Ethiopia, 2010-2013 (n=595)

Variables	Good treatment outcome (cure)	Poor treatment outcome	cOR (95% CI)	aOR (95% CI)
Age (yr)				
≥46	17	14	3.15 (1.01,9.90)	3.00 (0.80,10.50)
16-45	414	121	1.12 (0.45,2.80)	1.25 (0.45,3.50)
<15	23	6	1.00	1.00
Duration prior to diagnosis (d)				
≥29	76	65	5.25 (2.78, 9.90)	4.34 (2.20,8.46)^*^
14-28	286	61	1.30 (0.70, 2.40)	1.20 (0.60, 2.20)
<13	92	15	1.00	1.00
General condition				
Unable to walk	167	71	1.74 (1.20, 2.50)	1.63 (1.06, 2.40)^*^
Able to walk	287	70	1.00	1.00
Weight loss				
Yes	400	131	1.70 (0.80,3.50)	1.60 (0.70, 3.40)
No	54	10	1.00	1.00
Hepatomegaly				
Yes	15	9	2.00 (0.85, 4.66)	1.64 (0.60, 4.20)
No	439	132	1.00	1.00
Hemoglobin (g/dL)				
<7.9	210	69	1.40 (0.89, 2.14)	0.90 (0.50,1.45)
8.0-10.9	71	31	1.80 (1.07,3.16)	1.20 (0.64, 2.15)
≥11.0	173	41	1.00	1.00
Platelet count (cells/mm^3^)				
<85,000	188	77	1.60 (1.10, 2.40)	1.38 (0.87, 2.20)
≥85,000	266	64	1.00	1.00
Tuberculosis				
Yes	40	21	1.80 (1.03, 3.20)	1.39 (0.73, 2.67)
No	414	120	1.00	1.00
HIV/AIDS				
Yes	26	23	3.20 (1.70, 5.80)	2.72 (1.40, 5.20)^*^
No	428	118	1.00	1.00

cOR, crude odds ratio; aOR, adjusted odds ratio; CI, confidence interval; HIV, human immunodeficiency virus; AIDS, acquired immune deficiency syndrome.

* p<0.05.
